# Precursor B-Cell Lymphoblastic Lymphoma Presenting as a Spinal Mass at Initial Diagnosis

**DOI:** 10.4274/tjh.2015.0294

**Published:** 2016-05-16

**Authors:** Oğuzhan Erol, Çiğdem Tokyol, Feyzullah Akyüz, Nuran Ahu Baysal, Mehmet Sezgin Pepeler

**Affiliations:** 1 Afyon Kocatepe University Faculty of Medicine, Department of Pathology, Afyonkarahisar, Turkey; 2 Park Hospital, Clinic of Neurosurgery, Afyonkarahisar, Turkey; 3 Afyonkarahisar Public Hospital, Clinic of Hematology, Afyonkarahisar, Turkey; 4 Gazi University Faculty of Medicine, Department of Hematology, Ankara, Turkey

**Keywords:** B-cell lymphoblastic lymphoma, Thoracic spine, Spinal cord compression

An 18-year-old male presented to the emergency department of our hospital with complaints of bilateral leg numbness and weakness since about a month. Magnetic resonance imaging of the spine revealed an extramedullary extradural mass at the T9-T11 level causing marked spinal cord compression. Emergent surgery was performed. An epidural mass was seen after laminectomy and partially removed. Microscopic examination showed a diffuse infiltration of small- to medium-sized lymphoid cells with irregular nuclei, dispersed nuclear chromatin, prominent nucleoli, and scant cytoplasm in adipose tissue (Figure 1). Immunohistochemical examination demonstrated that tumor cells stained positively for TdT, CD34, CD10, CD20, CD79a, Pax-5, CD45, and Bcl-2 (Figure 2). Ki-67 showed immunoreactivity of 80% of tumor cells. Bone marrow and blood involvements were not detected. These findings led us to the diagnosis of precursor B-cell lymphoblastic lymphoma. He was given combination chemotherapy of R-HCVAD (rituximab, cyclophosphamide, vincristine, doxorubicin, dexamethasone, cytarabine, mesna, methotrexate). After the second dose of chemotherapy, complete response was achieved as assessed by positron emission tomography/computed tomography scan.

The spinal cord is an extremely rare initial site of involvement for B-cell lymphoblastic lymphoma. To our knowledge, there are only 3 reported cases in the English literature (Table 1) [1,2,3].

Lymphoblastic lymphoma should be included in the differential diagnosis of spinal masses.

## Figures and Tables

**Table 1:**
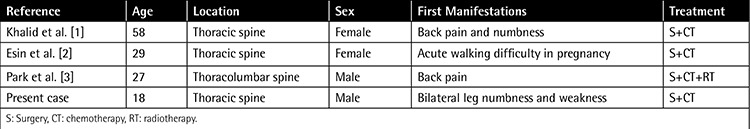
Cases of isolated primary B-cell lymphoblastic lymphoma of the spine.

**Figure 1:**
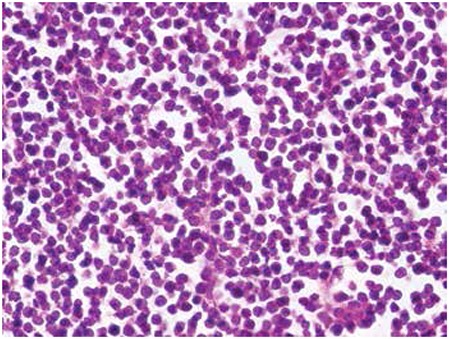
Lymphoid cells with irregular nuclei, dispersed nuclear chromatin, prominent nucleoli, and scant cytoplasm (H&E, 400x).

**Figure 2:**
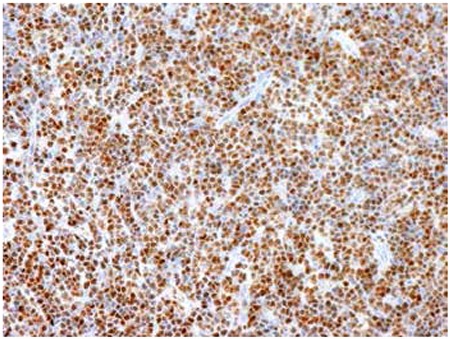
Diffuse expression of TdT in tumor cells (200x).
